# Evaluation of selected carotenoids of *Lycopersicon esculentum* variants as therapeutic targets for ‘Alzheimer’s disease: an in *silico* approach

**DOI:** 10.1186/s12860-021-00386-2

**Published:** 2021-10-01

**Authors:** Olalekan Olanrewaju Bakare, Adewale Oluwaseun Fadaka, Musa Oyebowale Akanbi, Kolajo Adedamola Akinyede, Ashwil Klein, Marshall Keyster

**Affiliations:** 1 Bioinformatics research group, Department of Biotechnology, Faculty of Natural Sciences, University of the Western Cape, Private Bag X17, Bellville, Cape Town, 7535 South Africa; 2 Environmental Biotechnology Laboratory (EBL), Department of Biotechnology, Faculty of Natural Sciences, University of the Western Cape, Cape Town, South Africa; 3 Department of Science and Technology/Mintek Nanotechnology Innovation Centre, Biolabels Node, Department of Biotechnology, Faculty of Natural Sciences, University of the Western Cape, Private Bag X17, Bellville, 7535 South Africa; 4 Department of Medical Bioscience, University of the Western Cape, Bellville, Cape Town, 7535 South Africa; 5 Plant Omics group, Department of Biotechnology, Faculty of Natural Sciences, University of the Western Cape, Private Bag X17, Bellville, Cape Town, 7535 South Africa

**Keywords:** Toxicity, Drug-likeness, Therapeutic, Inhibitors, Phytochemicals, Ligands, ‘Alzheimer’s, In silico, Carotenoids

## Abstract

The seriousness and menace of the worldwide weight of ‘Alzheimer’s disease have been related to a few factors, which incorporate antioxidant system depletion, mutation of proteins, and high expression of cholinesterases due to aging, environmental influence, diet, infectious agents, and hormonal imbalance. Overexpression of cholinesterases has been emphatically connected to ‘Alzheimer’s disease because of the unreasonable hydrolysis of acetylcholine and butyrylcholine. Certain plant phytochemicals, for example, beta-carotenoids, lutein, neoxanthin, and viola-xanthine from *Lycopersicon esculentum* Mill. Var. esculentum (ESC) and *Lycopersicon esculentum* Mill. Var. cerasiforme (CER) has been utilized altogether as a therapeutic candidate for the treatment of ‘Alzheimer’s disease. Therefore, this research sought to investigate the drug-likeness of the individual carotenoids as detailed for cholinesterase inhibition in the treatment of ‘Alzheimer’s disease. Four potential cholinesterase inhibitors from ESC and CER were retrieved from the PubChem database. Investigation of their drug-likeness, toxicity prediction, molecular docking, and dynamic simulations were carried out using Molinspiration, PreADMET V.2.0, Patchdock server, and Schrodinger Maestro software respectively. Neoxanthin was ranked the safest with a greater tendency to inhibit the cholinesterases with high binding affinity. In addition, its stability after simulation in a mimicked biological environment suggests its relevance as a potential drug candidate for the treatment of ‘Alzheimer’s disease through the inhibition of cholinesterases.

## Introduction

Fifty million people have ‘Alzheimer’s disease worldwide, according to the WHO reports, with approximately ten million cases each year [2]. This disease contributes 70% of the disability and dependency of older people, thus impacting not only on the psychological, physical, social, but economic aspects of caregivers, families, and societies at large [5]. It is chronic and progressive in nature, causing deterioration of the cognitive function of the brain, either primarily or secondarily, which can affect emotional control, social behaviour, and motivation [13]. However, the recent exponential increase in this disease across ages less than 65 years and associated burden in economically developing countries is becoming alarming [30]. The major contributing factors are that the economically developing countries lack the human resources, adequate screening centres, and ill-equipped to cope with the growing challenges of the disease [40]. Despite enormous efforts by researchers and international communities, the developing pace of the sickness is constantly expanding.

The exact mechanism by which ‘Alzheimer’s disease affects humans is not fully understood. It is believed that improper folding of tau proteins causing beta-amyloid plaques and neurofibrillary tangle, overproduction of acetylcholinesterase (AChE) and butyrylcholinesterase (BChE), resulting in low stimulation of neurons and depletion of the antioxidant system caused by the ‘brain’s radical-induced damage due to the low level of antioxidant enzymes and high dependency on oxygen are the major causes of the disease [27]. In the last few years, significant advances have been made regarding the treatment and prevention of this disease [27, 45]. The use of chemotherapy, such as donepezil and galantamine as cholinesterase inhibitors, which hitherto have been used to manage the symptoms of the disease, suffer some limitations such as toxicity, cell cycle specificity, and development of drug resistance [34]. These challenges pose serious threat to the management and cure of the disease, thus developing new molecules as alternative treatment of the disease is imperative.

Three approaches can enhance the treatment of ‘Alzheimer’s disease, namely: identification of therapeutic candidates from natural sources with inhibitory specificity that will leave the normal brain cells healthy with their development, novel vaccine development for prevention and control, and development of diagnostic tools for early detection [6]. The use of functional foods as AChE and BChE has been identified as alternative and cheap sources of therapeutic candidates without hepatotoxicity due to their antioxidant and other compensatory properties. Oboh et al. [35] used two *Lycopersicon esculentum* varieties extracts as AChE and BChE inhibitors for the dietary treatment of ‘Alzheimer’s disease, where some carotenoids such as lutein, neoxanthin, violaxanthin, and betacarotene, among others were reported to be very abundant in these plant extracts. According to the relative abundance of the GC-MS reports, the four carotenoids mentioned above were reported to be very abundant in these tomato variants. However, it was not stated the extent of these ‘carotenoids’ drug-likeness in the management of ‘Alzheimer’s disease.

The current lack of cure for Alzheimer’s disease poses a major challenge because many anticholinesterase drugs such as memantine only ameliorate the symptoms of the dementia and are not capable of preventing the progression of the disease [42, 50]. There is a huge limitation and set-back in the discovery of an effective drug candidate due to the failed clinical trials of the drugs. Attention is currently being focused on natural plant products for the management of the disease due to their low toxicity; while new research innovations are being developed to tailor these phytochemicals into therapeutic agents for neurodegenerative diseases [25, 42]. To this effect, the role of multi-target drug candidates for multi-functional Alzheimer’s disease using cholinesterase inhibition and others, for example N-methyl-D-aspartic acid receptor (NMDAR), as molecular targets has been explored [19, 20, 47]; however, several challenges which include undesirable side effects, incessant incidence of drug resistance, and reduced efficacy against diseases for which pathogenesis relies on many biochemical events and bioreceptors working concomitantly necessitate the need for the design of sensitive drugs with specific targets [31].

The use of in silico tools in drug formulations has become increasingly popular because they save time, are less labour intensive, and less expensive with high accuracy and specificity. These tools have been designed to incorporate certain properties which are said to be important for drug formulations. Examples of such properties include solubility, Ph, cytotoxicity, and the screening of impurities or degradants for mutagenicity. These in silico tools include ADMET predictor, Chemicalize online resource, PATCHDOCK, Molinspiration cheminformatics tool, just to mention a few. They are designed to have different functions, which, when used together, can enhance the discovery process by analyzing very large data without worrisome analytical testing. Such functions include a better understanding of the biological responses for the reduction of uncertainties in certain extrapolations and an allowance of prediction of treatment responses that consider ‘patients’ genetic differences or prior diseases.

Therefore, the aim of this present study was to investigate the drug-likeness and capacity of the four most abundant carotenoids present in two *Lycopersicon esculentum* varieties for the management of ‘Alzheimer’s disease. These would help proffer a solution to the menace of ‘Alzheimer’s disease caused by the overexpression and dysregulation of AChE and BChE, which could subsequently serve as a corrective measure to the degree of alteration and mutation of proteins at the neuromuscular junction. The use of in silico tools would reduce the time and enhance and the quality of the drug discovery. The information gathered from this analysis would guide researchers on the best means to utilize these vital compounds, whether as dietary or from the isolation of each carotenoid compound in the form of a drug for the treatment of the disease.

## Methods

### Collection of bioactive compounds

From our previous study, the four carotenoid compounds which were identified in ESC and CER using GC-MS were further investigated for their inhibitory role against AChE and BChE for the treatment of ‘Alzheimer’s disease [35] with donepezil being used as standard cholinesterase inhibitor [4]. Briefly, beta-carotene, neoxanthin, violaxanthin, and lutein were retrieved from PubChem (https://pubchem.ncbi.nlm.nih.gov/) database in SDF format [26]. Conversion of the SDF files to SMILES files was then carried out using Open Babel Converter (http://openbabel.org/wiki/Main_Page).

### Lipinski’s rule of five investigation of the bioactive compounds

The ‘Lipinski’s rule of five was used to evaluate the ‘compounds’ drug-likeness with the aid of Molinspiration cheminformatics tool (https://www.molinspiration.com/cgi-bin/properties) that employs a supercomputing resource for biological computation bioinformatics analysis by calculating molecular properties, predicting biological activities, and virtual screening of the ligands [29].

### Prediction of toxicity

The toxicity analysis of the compounds was carried out using preADMET version 2.0 (https://preadmet.bmdrc.kr/preadmet-pc-version-2-0/) to predict their absorption, distribution, metabolism and elimination/excretion (ADME) properties and evaluate safety through in silico approach [28].

### Preparation of ligands and receptors

LigPrep (https://www.schrodinger.com/products/ligprep) was used to prepare the ligands for further docking analytical studies using Schrodinger Maestro which employs many force fields [36]. The generation of a solitary, reduced energy, and 3D structure with chiralities by optimizing geometries using Desalt, ring conformation, stereochemistries and tautomers, an indication of fruitful structure. Selection of a binding site in the target proteins was carried out prior to the commencement of molecular docking. A method by Fadaka et al. [16] was used to incorporate hydrogen atoms, eliminate alternate conformations and HetAtoms from proteins, add and correct the missing residues, and many more.

### Docking studies using PatchDock and Schrodinger maestro software

The docking analysis of the ligands and the receptors were carried out using two docking tools (Patchdock (https://bioinfo3d.cs.tau.ac.il/PatchDock/php.php) [44] and Schrodinger suit) in order to ensure accuracy according to our previous methods [18, 37]. Briefly, Patchdock server was used to evaluate the atomic contact energy, area, and the binding scores of the complexes while Schrödinger suit was employed to evaluate the binding interaction, dock score, as well as the Glide scores of the ligands against BChE and AChE. Visualization of the complexes was achieved by Discovery Studio Visualizer (DSV v.4.0).

### Calculation of the prime MM-GBSA

The receptor-ligand complex free binding energy was calculated using the molecular mechanically generalized Born surface area (MM-GBSA) [15, 24]. The binding free energy, relative distance between the complex and PIC50 values were generated.

### Molecular dynamics (MD) simulation of the best ligand candidate and the receptors

MD simulation was carried out using the Desmond module of Schrodinger programming with force field OPLS 2005 [14, 36]. Electrostatic interactions were determined, which computes the ‘ligand’s relative stability in the binding pockets of the receptors using RMSD plots for both the proteins and the ligand-bound proteins. The analysis of the results was visualized by the simulation interaction diagram and MS-MD trajectory analysis [1, 17].

## Results

### Retrieval of bioactive compounds

The four bioactive compounds of ESC and CER reported in our previous work [35] belonged to the carotenoid class of phytochemicals. Their chemical structures were retrieved from the PubChem database with a cholinesterase inhibitor, donepezil, retrieved as control (Table 1). The four bioactive compounds were obtained in SDF formats and were later converted to SMILES formats. The 3-D structures of the PDB files were visualized using BIOVIA Discovery Studio Visualizer (DSV) version 4.0 [3].

**Table 1:**
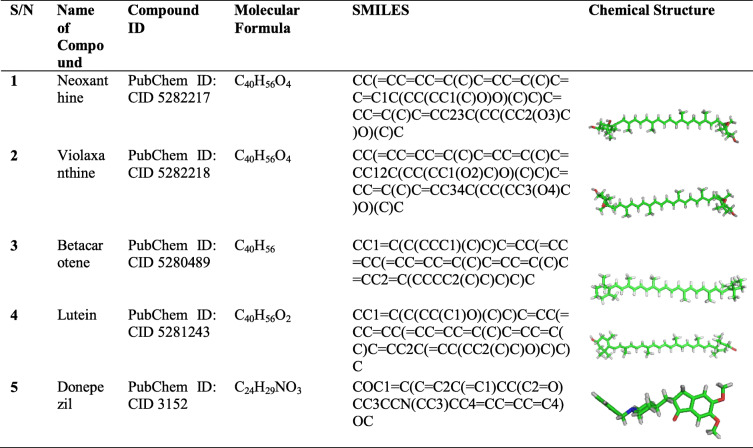
Bioactive Carotenoids of ESC and CER

### Lipinski’s rule of five (RO5) analysis

This analysis, also called ‘Pfizer’s rule, is used to investigate the drug-likeness of any chemical compound by determining whether the compound has a particular biological attribute or pharmacological relevance that would make it potential oral drug in humans [29]. Another importance of this analysis is the tendency to predict the possibility of success or failure of drug-likeness of molecules simply by fulfilling certain rules such as high lipophilicity depicted as LogP usually below 5, molecular mass not greater than 500 Da, hydrogen bond acceptors below 10, hydrogen bond donors below 5, and molar refractivity within the range of 40–130 [23, 29]. Table 2 shows that the four carotenoids did not satisfy all the five requirements, for instance, none of the ligands satisfied the molecular mass which should not be greater 500 Da except donepezil and the ligands also had high lipophilicity greater than 5. Several mechanisms exist to efficiently impact the candidacy of drugs with high molecular weight and high lipophilicity for their proper delivery and bioavailability as oral drug candidates such as nanocomminution [8, 10] and high molecular weight drugs have been associated with good stability when compared to low molecular grade polymers [7]. The ligands possessed certain compensatory outcomes for hydrogen bond donors, hydrogen bond acceptors which impact on their stability, properties, and molecular functions [52] whilst excellent molar refractivity values are necessary for the efficient control of dispersive forces in drug-receptor interaction [39]. Interestingly, donepezil satisfied all the rules except very low result for molar refractivity.

**Table 2:** Evaluation of the Drug-likeness of the compounds using Lipinski Rule of Five

S/N	Compounds	Molecular Weight (Da)	Hydrogen bond donors	Hydrogen bond acceptors	milogP Values	Molar Refractivity
1	Neoxanthine	600,88	3	4	8.70	73.21
2	Violaxanthine	600.88	2	4	8.99	65.51
3	Betacarotene	536.89	0	0	9.84	40
4	Lutein	568.89	2	2	9.31	42
5	Donepezil	379.50	0	4	4.10	38.78

### Toxicity prediction of the bioactive compounds

Table 3 reveals neoxanthin, violaxanthin, betacarotene, lutein, and donepezil to be non-carcinogenic and they posed a medium risk in their ability to inhibit HERG. This result indicates varying genetic expression in their ability to be non-carcinogenic [38]. This indicates the safety of the ligands for consumption. Biological/pharmacological activities of the five carotenoids were further computed where the blood brain barrier partition coefficient result revealed that beta-carotene had the highest value (25.995) whereas donepezil had the lowest (0.188). Donepezil had the highest pure water solubility property (6.236 mg/L) with lutein having the lowest value (0.003). All the carotenoids had significant skin permeability values except for donepezil (− 3.042); the carotenoids also had significant human intestinal absorption values with beta-carotene having the highest (100.000). All the carotenoids had high plasma protein binding values with donepezil scoring lowest (84.616). The tendency for buffer solubility also revealed lutein with the lowest value of 0.002.

**Table 3:** Toxicity Prediction results of the bioactive compounds using PreADMET Web server

S/N	Compounds	Carcinogenic test (Mouse)	HERG Inhibition	BBB	PWS (mg/L)	SP	HIA	PPB	BS
1	Neoxanthine	Negative	Medium risk	9.010	0.020	− 0796	95.175	92.807	0.0549
2	Violaxanthine	Negative	Medium risk	9.300	0.007	−0.806	96.428	91.271	0.918
3	Betacarotene	Negative	Medium risk	25.995	2.646	−0.609	100.000	100.000	3.363
4	Lutein	Negative	Medium risk	16.260	0.003	−0.648	95.521	100.000	0.002
5	Donepezil	Negative	Medium risk	0.188	6.236	−3.042	97.951	84.616	5.101

Legend: *BBB* Blood Brain Barrier partition coefficient, *PWS* Pure Water Solubility, *SP* Skin Permeability, *HIA* Human Intestinal Absorption, *PPB* Plasma Protein Binding, and *BS* Buffer Solubility

### Molecular docking of the receptors and the ligands

3-D structure prediction interaction analysis was carried out using the Patchdock server. The binding scores, interface area, and atomic contact energy (ACE) of the complexes were displayed in Table 4. Patchdock produced higher binding scores for all the ligands than donepezil against AChE and BChE with beta-carotene having the highest binding score (AChE = 6626, BChE = 6548) which also ranked highest in interface area (AChE = 856.60, BChE = 957.20) and lowest atomic contact energy (ACE) (AChE = − 266.95, BChE = − 497.92) against both AChE and BChE. However, further interaction analysis was required as Patchdock results only showed the interaction between receptors and ligands but not the degree of stability in the receptors [12].

**Table 4:** Patchdock Analysis of Acetylcholinesterase (AChE) and Butyrylcholinesterase (BChE) and the Carotenoids

S/N	Compounds	Binding score	Area	ACE
1	Neoxanthine- AChE	6196	763.00	− 116.24
2	Violaxanthine-AChE	5660	686.90	−85.62
3	Betacarotene- AChE	6626	856.60	−266.95
4	Lutein- AChE	6502	784.40	− 226.07
5	Donepezil-AChE	5536	646.60	−202.36
6	Neoxanthine-BChE	6086	701.00	− 338.06
7	Violaxanthine- BChE	6216	770.60	− 387.56
8	Betacarotene- BChE	6548	957.20	−497.92
9	Lutein- BChE	6512	807.60	− 461.72
10	Donepezil- BChE	5186	629.70	− 212.23

The generated grid box and the ligand structure were used for docking analysis of the receptors BChE and AChE after optimizing the ligands utilizing the glide module in the Schrodinger suit. Table 5 showed neoxanthin having the best docking scores with binding scores of − 9.1 and − 10.2 against BChE and AChE respectively. No interaction was reported for Beta-carotene and lutein against AChE with violaxanthin having no interaction for BChE. Only donepezil had higher energy than neoxanthin against AChE and BChE. Neoxanthin formed three stable hydrogen bonding with three amino acids in both AChE (Asp_74, Tyr_72, and His_447) and BChE (Ile_69, Ser_72, and Tyr_332) (Fig. 2), whereas donepezil only formed three stable hydrogen bonds with AChE but none with BChE. The tendency of neoxanthin to form more stable hydrogen bonds with the receptors would impact the stability and molecular functions better than other ligands and donepezil [52].

**Table 5:** Binding affinities (KJ/mol) of the ligands against acetylcholinesterase and butyrylcholinesterase receptors calculated with Glide G score, Dock score, and MM/GBSA

		Acetylcholinesterase (AChE)				Butyrylcholinesterase (BChE)			
S/N	Compound	Glide score	DOCK SCORE	MM-GBSA	H-Bond	Glide score	DOCK SCORE	MM-GBSA	H-Bond
1	**Donepezil**	−16.6	−16.6	− 81.35	3	−8.9	−8.9	−50.25	0
2	B-carotene	*	*	*	1	−5.3	−5.3	−30.24	1
3	**Neoxanthin**	**−10.2**	**−10.2**	**− 103.11**	**3**	**−9.1**	**−9.1**	**−95.11**	**3**
4	Violaxanthin	−9.1	− 9.1	−103.43	1	*	*	*	*
5	Lutein	*	*	*	*	−5.2	−5.2	− 83.23	1

**Fig. 1:**
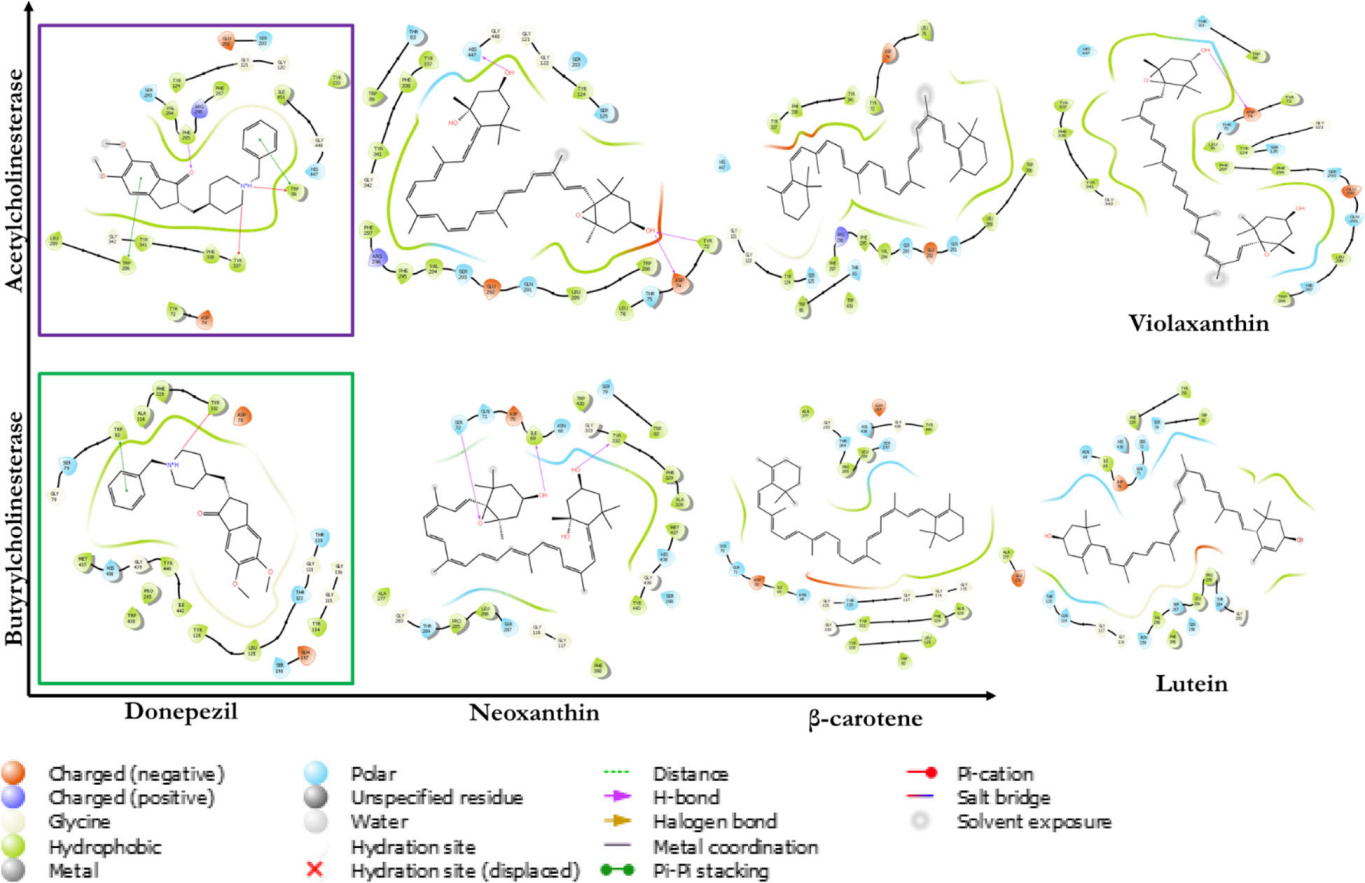
2D docking interaction studies of the ligands and receptors. Donepezil, neoxanthin, and beta-carotene interacted with both AChE and BChE while violaxanthin and lutein interacted with AChE and BChE respectively

Also shown in Fig. 1 below is the 2D interaction analysis of the ligands and donepezil with the receptors with all complexes displayed forming non-covalent bonding, an important consideration for drug development and discovery. Higher binding affinity could be observed for neoxanthin against the receptors with violaxanthin and lutein having no interaction with AChE and BChE, respectively.

**Fig. 2:**
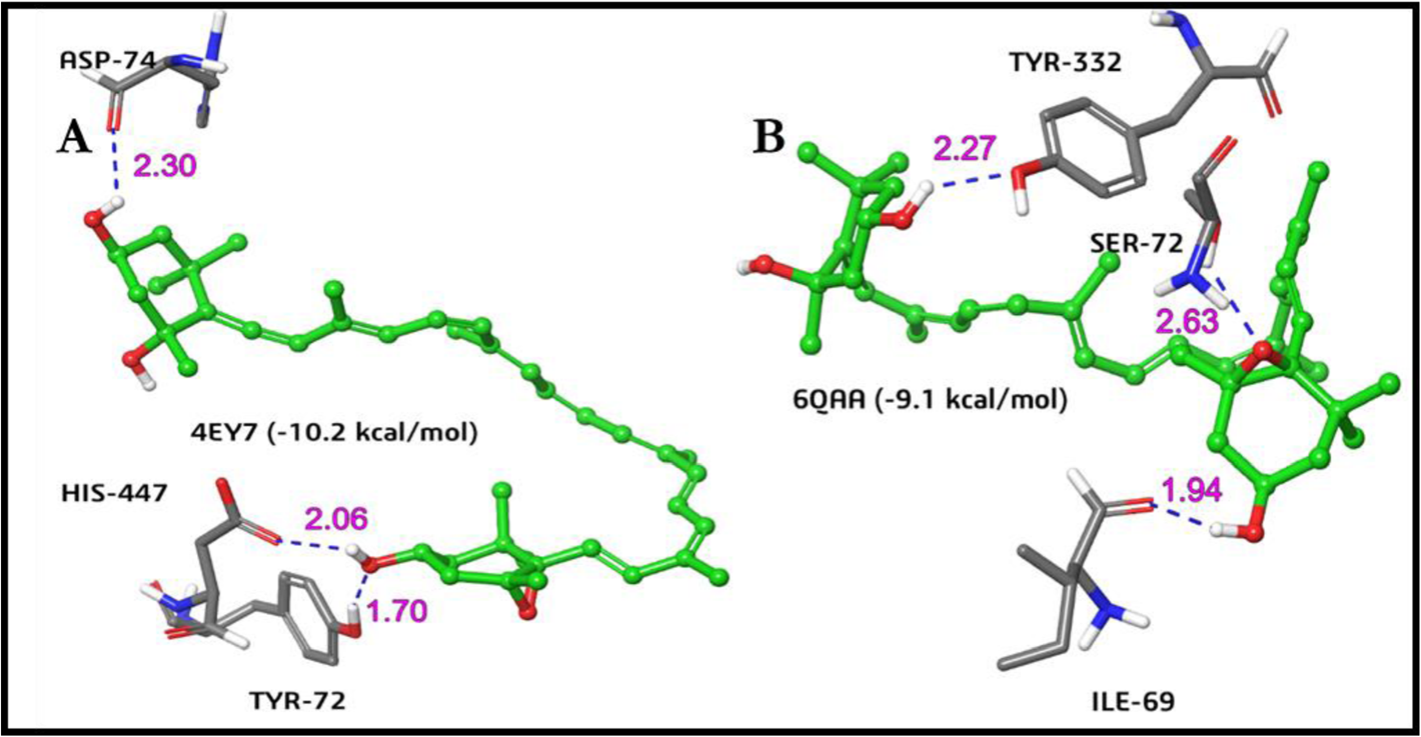
The docking analysis of Neoxanthin in the active sites of **A** acetylcholinesterase and **B** butyrylcholinesterase. Hydrogen bonds are represented with blue dotted line with their respective bond distance (pink) in Å

Neoxanthin also formed higher hydrogen bonding with AChE (Asp74, Tyr72, and His447) and BChE (Ile69, Ser72, and Tyr332) with relative distances as displayed in Fig. 2. Hence, neoxanthin was therefore selected for molecular dynamics simulation since it has proven more effective in its interaction with the cholinesterases with better stability than donepezil.

### Molecular dynamic simulation

**Fig. 3:**
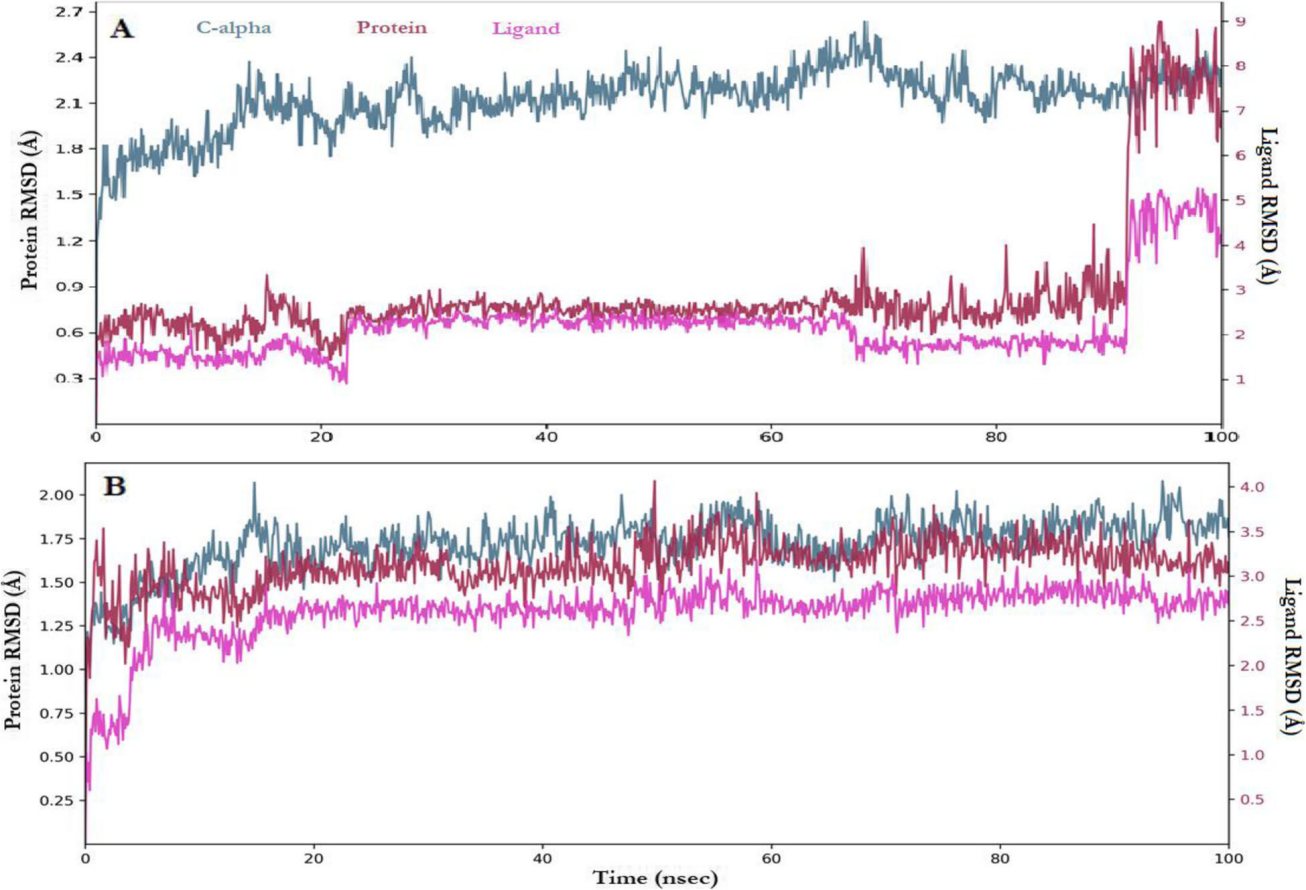
Ligand RMSD (Å) for neoxanthin against the cholinesterases AChE (**A**) and BChE (**B**) where the pink colour indicate ligand fluctuation with the receptors for the target of the binding site and the brown indicates fluctuation with the receptors for the alignment with the reference frame of the ligand

**Fig. 4:**
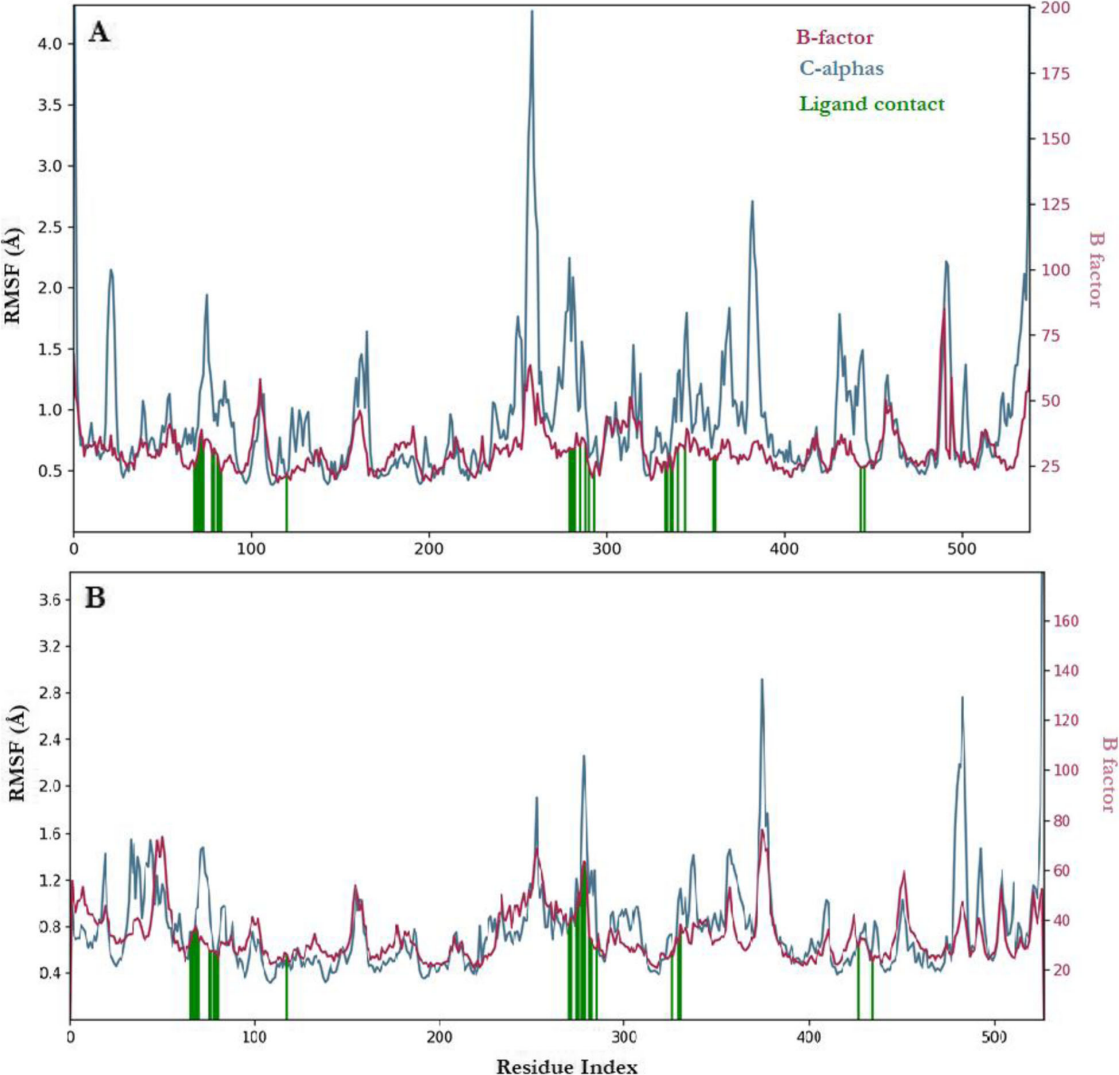
Protein RMSF interaction with neoxanthin against the cholinesterases AChE (**A**) and BChE (**B**) shown in code with green indicating the interaction of neoxanthin atoms with the receptors

**Fig. 5:**
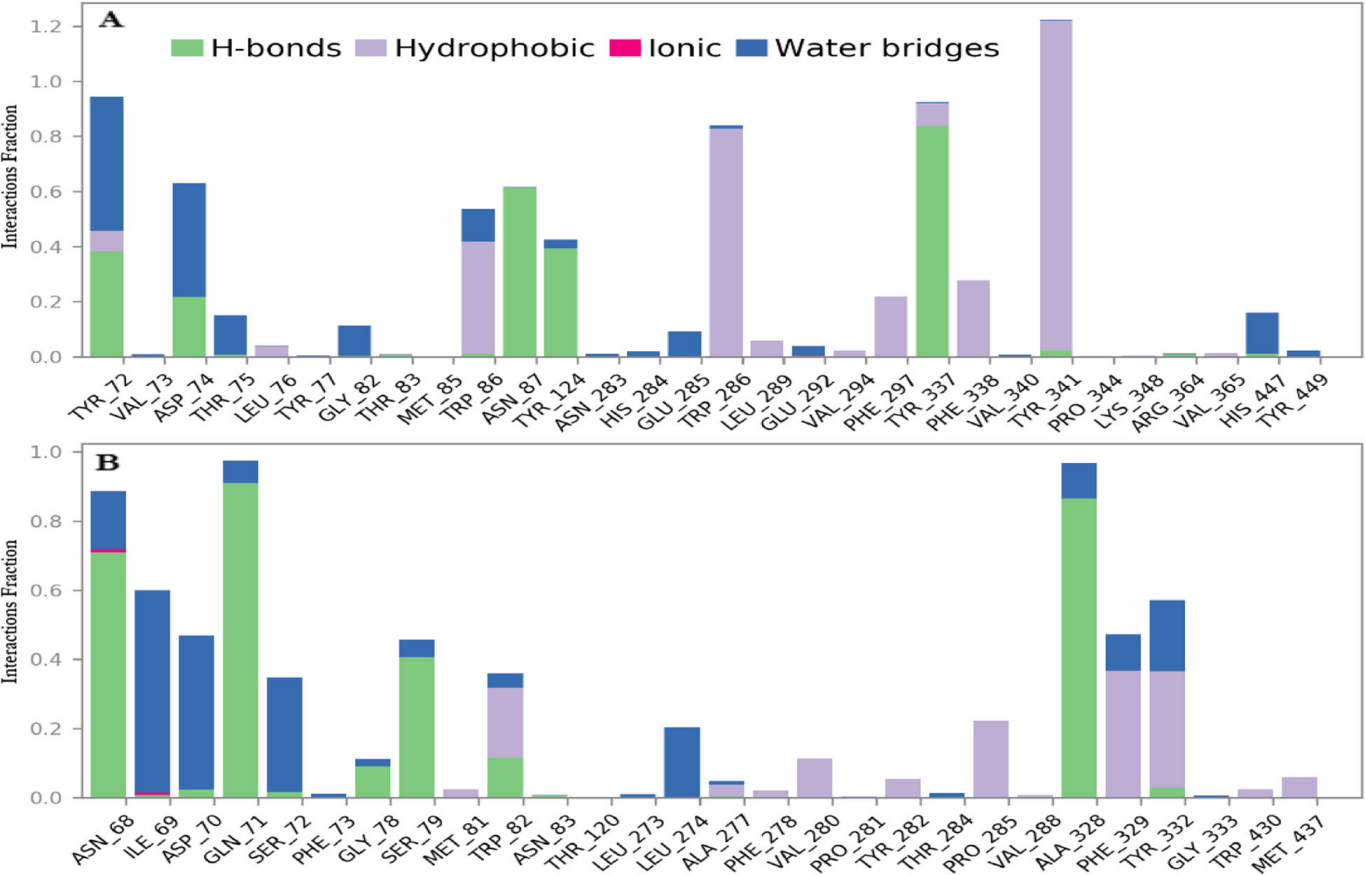
The histogram chart of interactions of neoxanthin-receptor complexes with AChE (**A**) and BChE (**B**) forming hydrogen bond, ionic bond, water bridg, and hydrophobic interactions using MD simulations

**Fig. 6:**
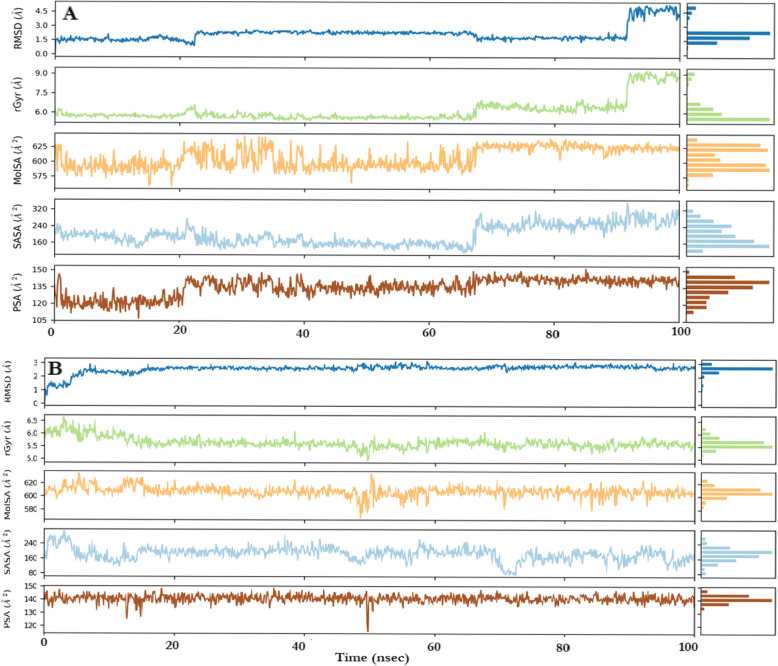
Fluctuation in neoxanthin properties with the complexes formed with acetylcholinesterase (**A**) and Butyrylcholinesterase (**B**) using root mean square deviation (RMSD), radius of gyration (rGyr), intramolecular hydrogen bonds (intraHB), molecular surface area (MolSA), solvent accessible surface area (SASA), and polar surface area (PSA)

The MD simulation was carried out for neoxanthin against the receptors being the ligand with the best inhibitory activity and was monitored for 100 nsec utilizing four interactions namely; hydrogen bonds, hydrophobic interactions, ionic bonds, and salt bridges (Figs. 3, 4, 5, and 6). In Fig. 3, the neoxanthine root mean square deviation (RMSD) plot indicates the stability with respect to the proteins and its binding pockets throughout the entire period. The ‘Lig fit Prot’ shows the RMSD of neoxanthin aligned on AChE and BChE backbones. Since the ‘Lig fit Lig’ showed the RMSD of neoxanthin was lower than the AChE and BChE significantly, then it means the neoxanthin had not diffused away from its initial binding sites.

Figure 4 below showed the interaction of neoxanthin with AChE (A) and BChE (B) where the interaction residues were depicted in green colour and the receptor secondary structures in terms of helices and β-strands are indicated with orange and blue bands.

In Fig. 5 below, neoxanthin displayed favoured water bridge interaction with the nitrogen atoms of the amino acids for AChE (A) at Tyr_72, Asp_74, Thr_75, Gly_82, Trp_86, Tyr_124, His_284, Glu_285, Glu_292, and His_447 whilst neoxanthin-BChE complex (B) had favoured water bridge interaction at Asn_68, Ile_69, Asp_70, Gln_71, Ser_72, Gly_78, Ser_79, Trp_82, Leu_274, Ala_277, Ala_328, Phe_329, and Tyr_332. Hydrogen bonding interaction was also observed for neoxanthin-AChE at Tyr_72, Asp_74, Asn_87, Tyr_124, Tyr_337, and Tyr_341 with the strongest and most stable ones formed for (Asp_74, Tyr_72, and His_447) whilst neoxanthin-BChE had hydrogen bonding at Asn_68, Asp_70, Gln_71, Ser_72, Gly_78, Ser_79, Trp_82, Ala_328, and Tyr_332 with the strongest and most stable ones formed for (Ile_69, Ser_72, and Tyr_332). Hydrophobic interaction was observed for neoxanthin-AChE complex at Tyr_72, Leu_76, Trp_86, Trp_286, Leu_289, Val_294, Phe_297, Tyr_337, Phe_338, and Tyr_341 whilst neoxanthin-BChE complex had hydrophobic interaction at Met_81, Trp_82, Ala_277, Phe_278, Val_280, Tyr_282, Pro_285, Phe_329, Tyr_332, Trp_430, and Met_437. Ionic interaction was observed for neoxanthin-BChE complex at Asn_68 and Ile_69 (Table 6).

**Table 6:** Bonding interactions displayed by neoxanthin against Acetylcholinesterase (AChE) and Butyrylcholinesterase (BChE)

Bonding interactins	AChE amino acid residues with positions	BChE amino acid residues with positions
Favoured water bridge interaction with nitrogen atom	Tyr_72, Asp_74, Thr_75, Gly_82, Trp_86, Tyr_124, His_284, Glu_285, Glu_292, and His_447	Asn_68, Ile_69, Asp_70, Gln_71, Ser_72, Gly_78, Ser_79, Trp_82, Leu_274, Ala_277, Ala_328, Phe_329, and Tyr_332
Hydrogen bonding interaction	Tyr_72, Asp_74, Asn_87, Tyr_124, Tyr_337, and Tyr_341	Asn_68, Asp_70, Gln_71, Ser_72, Gly_78, Ser_79, Trp_82, Ala_328, and Tyr_332
Hydrophobic interaction	Tyr_72, Leu_76, Trp_86, Trp_286, Leu_289, Val_294, Phe_297, Tyr_337, Phe_338, and Tyr_341	Met_81, Trp_82, Ala_277, Phe_278, Val_280, Tyr_282, Pro_285, Phe_329, Tyr_332, Trp_430, and Met_437
Ionic Interaction	–	Asn_68 and Ile_69

The interaction of the amino acid residues of the cholinesterases and the neoxanthin, as displayed in the Fig. 6, was responsible for the overall stability of the ligand using the different bond parameters.

Figure 6 below showed the molecular properties of neoxanthin receptor complexes using RMSD, radius of gyration (rGyr), intramolecular hydrogen bonds (intraHB), molecular surface area (MolSA), solvent accessible surface area (SASA), and polar surface area (PSA) carried out over 100 ns. The RMSD of the ligand indicated fluctuation over time; rGyr indicated neoxanthin extendedness using the principal moment of inertia; intraHB indicated the presence of internal bonds within neoxanthine; MolSA indicated the presence of van der Waal surface area; SASA indicated the presence of surface area accessible by water molecule; and the PSA showed that the solvent accessible surface area contributed by nitrogen and oxygen. In summary, all these properties were present for neoxanthin, indicating no large change was found for neoxanthin-AChE and neoxanthin-BChE.

## Discussion

In an attempt to enhance the management of ‘Alzheimer’s disease, many researchers have carried out studies to support the therapeutic intervention to manage and treat the disease. One of the major successes is the suppression or inhibition of AChE and BChE to delay the breakdown of acetylcholine into the synaptic cleft [41]. In line with the previous study, inhibition of these cholinesterases using carotenoids from ESC and CER has been utilized as an alternative therapy for ‘Alzheimer’s disease [35]. The knowledge about using natural phytochemicals as drugs is becoming important because of their safety and minor/no side effects [9]. This research, therefore, attempts to test and screen the anticholinesterase efficacy of four carotenoids found in ESC and CER for the treatment of ‘Alzheimer’s disease. Several carotenoids have been described to inhibit AChE and BChE, traverse the blood-brain barrier and possess antioxidant properties [33, 35]. This extraordinary potential has made them therapeutic candidates against neurodegenerative diseases, most especially ‘Alzheimer’s disease. With the use of in silico technology, the determination of the best therapeutic agent from the four carotenoid compounds with inhibitory efficacy against cholinesterases could serve as a potential drug with reduced toxicity, using donepezil as an index.

The use of in silico tools for biological analysis is becoming invaluable due to their importance in shortening the formulation period of novel drug candidates for clinical application and the tendency to reduce cost [48]. Appropriate drug target is fundamental to the drug discovery process. In contrast, validation of such a process is necessary to ascertain a level of confidence in the pharmacological potency of the disease [32]. During drug discovery and design of a candidate compound, several intensive tests such as oral bioavailability using tools such ‘Lipinski’s rule of five and toxicity test using tools such as PreADMET version 2,0, among others. These facilitate the early advancement in preclinical design and it also helps to avoid clinical failures and reduces cost in the late stage of the preclinical trial [24, 25]. All four compounds (lutein, neoxanthin, violaxanthin, and betacarotene) satisfied only three of ‘Lipinski’s rule of five with their molecular weight and lipophilicity out of range. It is also interesting to see that even donepezil, a standard anticholinesterase also violates one of the rules. It is believed that with the use of certain biomedical and pharmacological technologies such as nanocomminution, the utilization of high molecular weight and high lipophilic compounds as potent drugs is feasible for stability and bioavailability [8, 10].

Apart from this, the toxicity test for the compounds were also carried out in animal models with consideration to their carcinogenic potential; interestingly, these compounds share the same non-toxicity properties with donepezil, a standard drug for the amelioration of the disease. The pharmacological/biological activities of the carotenoids revealed significant blood brain barrier partition coefficient, pure water solubility, skin permeability, human intestinal absorption, plasma protein binding, and buffer solubility, with donepezil scoring lowest in its capacity to cross the blood brain barrier, bind plasma protein, and permeate the skin. These properties are very necessary for central nervous system activities and bioavailability. Lutein was a major concern in its capacity to dissolve in appropriate buffer. This result is in line with the work of Harika et al. [21] where docking study of benzimidazole was carried out using these pharmacological activities. However, it is still important to evaluate the sensitivity test of these carotenoids for their capacity to bind AChE and BChE targets specifically despite their excellent biological activities. For this reason, docking interaction analysis of the five bioactive compounds against AChE and BChE (Table 4) using Patchdock revealed that the ligands possessed higher binding affinity than donepezil, with the highest value recorded for beta-carotene. Table 4 showed the area of interaction for the compounds against the cholinesterases with the highest value recorded for beta-carotene. It was also observed that the lowest ACE values against AChE and BChE were recorded for betacarotene. However, the Patchdock result only displayed interaction between ligand and receptor without showing the extent of inhibition of the ligands on the receptors and the site of inhibition [12].

Furthermore, the anticholinesterase efficacy of the ligands was carried out where the molecular docking using the Schrodinger Maestro suit identified the ligand-binding pockets (sites) and conformation of the targets in the receptors using donepezil (a cholinesterase inhibitor used to manage ‘Alzheimer’s disease) (Table 5). Neoxanthin formed hydrogen bonding with the cholinesterases at three unique locations respectively with the three unique amino acids, whereas donepezil formed three unique bonds for acetylcholinesterase only. This unique property is important for polar activation, ligand affinity, and folding of the receptors, which justified the use of neoxanthin as a cholinesterase inhibitor [43, 49]. Docking analytical study using donepezil and the ligands against the receptors with Schrodinger Maestro suit showed that only donepezil and neoxanthin had appreciable anticholinesterase activities against the receptors with the lowest glide and binding scores observed for donepezil and neoxanthin, respectively. However, the only neoxanthin had a much lower molecular mechanically generalized Born surface area (MM-GBSA) than donepezil. Several studies have reported donepezil as a standard drug for managing ‘Alzheimer’s disease through cholinesterase inhibition [46]. The high docking energy, glide energy, and molecular mechanically generalized Born surface area (MM-GBSA) scores of neoxanthin could be employed for rational drug discovery in the management of ‘Alzheimer’s dementia.

Experimental evidence exists to strengthen further the results from this study that carotenoids can reduce the incidence of Alzheimer’s disease with oxidative stress in the brain being said to cause a range of problems, including cognitive decline and onset of Alzheimer’s disease. A diet high in antioxidants, including vitamin C, vitamin E, and carotenoids such as beta-carotene, may aid in minimizing the incidence of Alzheimer’s disease, according to several population studies [22]. There is also evidence that lutein can pass the blood-brain barrier. Multiple studies showed lutein to be strongly linked to age-related cognitive decline and the risk of Alzheimer’s disease in humans [51]. However, cholinesterase inhibitors, which improve acetylcholine availability at cholinergic synapses, are currently the main cornerstone therapies for Alzheimer’s disease [11]. This study, therefore, explored the anticholinesterase inhibition of lutein, betacarotene, neoxanthine, and violaxanthine for the treatment of Alzheimer’s disease. This in silico study corroborated the previous experimental studies that carotenoids can reduce the incidence of cognitive impairment such as Alzheimer’s disease, with neoxanthine having better performance as a cholinesterase inhibitor than the other carotenoids used in this study. This is the first study that compared and evaluated the performance of these carotenoids using their anticholinesterase rather than antioxidant properties.

The significance of this study lies in the direction of a fresh route that could help in Alzheimer’s treatment development. This is because the knowledge that neoxanthin and other carotenoids could inhibit cholinesterases more effectively from a natural source with conformational stability that ensured minimal energy was dissipated throughout the whole system for assurance flexibility towards handling during drug formulation using relevant stereochemistry. The identification of AChE and BChE as targets for the carotenoids was very essential for the overall drug development success. From this finding, it could be inferred that neoxanthin would make a good adjuvant for drug formulation against ‘Alzheimer’s disease using anticholinesterase mechanism, and our result is significant because it is the first in silico study of the bioactive compounds present in *Lycopersicon esculentum* against AChE and BChE for the amelioration of ‘Alzheimer’s disease.

## Conclusion

This research work used in silico technologies to screen several carotenoids in terms of their druggable potency towards inhibition of cholinesterases to ameliorate ‘Alzheimer’s disease. Despite significant biological/pharmacological potency and safety exhibited by these carotenoids more than donepezil, most especially beta-carotene, neoxanthin had specific docking interaction at the binding sites expected for the inhibition of the cholinesterases. The outcomes of this finding showed that neoxanthin could serve as a drug-likeness against cholinesterases in the management of the disease. It is noteworthy to add that neoxanthin can be used in the formulation of diet supplements or as a standalone drug using the technologies used in this research for the intervention of public health concerns caused by the disease. Thus, its utility as an individual oral drug design is safe and efficacious against ‘Alzheimer’s disease.

### Future work

Future work would incorporate the in vivo *and* in vitro studies such as cytotoxicity studies and molecular validation assays to further establish the significance of the compounds relative to these in silico studies for corroboration of safety, proper delivery and bioavailability as oral drug candidates. Clinical trials in human beings would follow to establish the molecular dynamics simulation reports.
